# Rapid visual engagement in neural processing of detailed touch interactions

**DOI:** 10.1162/IMAG.a.1017

**Published:** 2025-11-17

**Authors:** Sophie Smit, Almudena Ramírez-Haro, Genevieve L. Quek, Manuel Varlet, Denise Moerel, Tijl Grootswagers

**Affiliations:** The MARCS Institute for Brain, Behaviour and Development, Western Sydney University, Sydney, Australia; School of Psychology, Western Sydney University, Sydney, Australia; School of Computer, Data and Mathematical Sciences, Western Sydney University, Sydney, Australia

**Keywords:** observed touch perception, rapid visual processing, affective touch, tactile features, electroencephalography, multivariate decoding

## Abstract

Touch perception is an inherently multisensory process in which vision plays an essential role. However, our understanding of how vision encodes sensory and emotional-affective aspects of observed touch, and the timing of these processes, remains limited. To address this gap, we investigated the neural dynamics of visual touch perception using electroencephalographic (EEG) recordings from participants who viewed videos depicting detailed tactile hand interactions from the Validated Touch-Video Database. We examined how the brain encodes basic body cues, such as hand orientation and viewing perspective, in addition to sensory aspects, including the type of touch (e.g., stroking vs. pressing; hand vs. object touch) and the object involved (e.g., knife, brush), as well as emotional-affective dimensions. Using multivariate decoding, we found that information about body cues emerged within approximately 60 ms, with information about sensory details and valence emerging around 110–160 ms, demonstrating efficient early visual encoding. Information about arousal, threat, and pain was most clearly identified by approximately 260 ms, suggesting that such evaluations require slightly extended neural engagement. Frequency decoding revealed that body cues were processed across a broad spectral range, with strongest contributions in the theta, alpha, and low beta bands (~6–20 Hz), while sensory and emotional-affective features were primarily reflected in delta, theta, and alpha frequencies (~1–13 Hz). Our findings reveal that bottom-up, automatic visual processing is integral to complex tactile assessments, important for rapidly extracting both the personal relevance and the sensory and emotional dimensions of visually observed touch.

## 1 Introduction

Touch is a fundamental channel of social and emotional communication. It shapes infant development, strengthens bonds between partners and peers, and conveys safety, threat, and intention in everyday interactions (Gallace & Spence, 2010; McGlone et al., 2014). Visual cues are central to this process, with evidence showing that even infants exhibit early neural responses to observed touch and pain, including mirror-touch experiences (Addabbo et al., 2020, 2021; Meltzoff et al., 2018). Understanding how the brain processes visual information about touch, therefore, offers insight not only into visual and multisensory perception, but also into the core mechanisms of social cognition.

When visually observing touch, our brain processes visual body cues, such as the location of touch and whether it involves our body or another’s—indicated, for instance, by first- or third-person perspectives—and sensory and affective information, like the pressure, texture, and emotional tone of the touch (Adler & Gillmeister, 2019; Boehme et al., 2019; Bufalari & Ionta, 2013; Butti et al., 2024; Gallace & Spence, 2010; Gazzola et al., 2012; Lamm et al., 2015; Lee Masson & Isik, 2023; Lee Masson et al., 2018, 2020; Peled-Avron & Woolley, 2022; Rigato et al., 2019b; Schirmer & McGlone, 2019; Smit et al., 2019, 2025a). It might seem that processing this array of diverse information requires complex, high-level computations. Yet, evidence increasingly shows that recognition of social and affective cues in human interactions primarily relies on rapid, automatic, bottom-up visual processes, suggesting an evolutionarily adaptive mechanism (McMahon & Isik, 2023; McMahon et al., 2023; Pitcher & Ungerleider, 2021; Scholl & Gao, 2013). Recent theories propose that understanding social interactions, including their valence, goals, intent, and possibly even the type of interaction, is a fundamentally visual process (McMahon & Isik, 2023). This does not imply mere low-level processing followed by cognitive interpretation, but rather suggests that our visual system contains advanced, abstract representations of social interactions. In observing social interactions that involve touch, such as two people hugging, dimensions like valence and arousal are processed within 180 ms (Lee Masson & Isik, 2023). This highlights the brain’s capacity to rapidly extract the social-affective meaning of touch through feedforward visual processing (Lamme & Roelfsema, 2000), emphasizing social touch interpretation as an integral aspect of visual perception. However, the encoding of other affective aspects, such as threat and pain, in visual touch perception remains poorly understood. Likewise, the timing of such representations relative to other tactile features, such as whether the touch involves an object or direct skin contact, or distinctions like pressing versus stroking, also remains unclear. To address this gap, we investigated the neural dynamics of visually observed detailed touch interactions, examining how the brain encodes body cues, sensory features, and emotional-affective dimensions.

Research on the neural basis of visually observed touch has often centred on the involvement of the somatosensory cortex (for reviews see Bufalari & Ionta, 2013; Gillmeister et al., 2017; Keysers & Gazzola, 2009; Peled-Avron & Woolley, 2022), an area of the brain responsible for processing direct tactile inputs. Such research shows that visually observing touch can activate the somatosensory cortex both at early sensory and later cognitive stages (Adler & Gillmeister, 2019; Adler et al., 2016; Bufalari et al., 2007; Galilee & McCleery, 2016; Martínez-Jauand et al., 2012; Pihko et al., 2010; Rigato et al., 2019a, 2019b; Smit et al., 2025a; Streltsova and McCleery, 2014). Such findings have been taken to suggest that we internally replicate the sensory experiences observed in others as though the touch were happening to us, facilitating a form of ‘tactile empathy’ (Lamm et al., 2015; Marsh, 2018). This aligns with theories like those of mirror neurons (Gallese & Goldman, 1998; Keysers & Gazzola, 2009). However, others have questioned the idea that somatosensory simulation is necessary for understanding others’ tactile experiences, proposing instead that visual processing alone may suffice (de Vignemont, 2017; Hickok, 2014). This raises an intriguing possibility: that the brain might extract sensory and emotional information from observed touch purely through visual pathways, before potentially engaging somatosensory regions. If this is the case, we would expect information about these dimensions of observed touch to emerge during the early stages of stimulus processing, within the first 150–200 ms. Previous research has largely focused on visually-induced somatosensory activity, leaving a significant gap in our understanding of how purely visual processes may encode the detailed sensory and emotional-affective aspects of perceived touch.

Oscillatory brain activity may provide important insight into how the brain processes visually observed touch. Slower brain rhythms, particularly delta and theta, have been associated with functions potentially relevant to visual touch perception, including emotion-related visual processing (Güntekin & Başar, 2014; Knyazev, 2007, 2012) and vicarious experiences of affectionate touch (Schirmer & McGlone, 2019). Alpha-band activity has also been linked to vicarious sensory perception (Peled-Avron et al., 2016; Whitmarsh et al., 2011; Yang et al., 2009), and to attentional focus on directly experienced tactile stimuli (Whitmarsh et al., 2017). Moreover, alpha activity appears to support internal visual representations during mental imagery (Arnold et al., 2024; Stecher & Kaiser, 2024; Xie et al., 2020). Despite these associations, it remains unclear how different frequency bands contribute to the encoding of body-related, sensory, and emotional dimensions of observed touch. Clarifying these frequency-specific mechanisms could deepen our understanding of the neural systems underlying visual touch perception.

In the current study, we examined the neural dynamics of visual touch perception using high-temporal resolution electroencephalography (EEG) as participants viewed close-up touch interactions from the Validated Touch-Video Database (Smit & Rich, 2025). We analyzed both the timing of these processes and the contributions of different frequency bands to the encoding of touch-related information. Based on prior work, we expected body cues to be represented earlier in the EEG signal (e.g., perspective; Rigato et al., 2019b), with sensory and emotional-affective dimensions emerging later, though still within the initial feedforward sweep (Fan & Han, 2008; Lee Masson & Isik, 2023). Using multivariate whole-brain decoding, we found that sensory and emotional details, such as the object involved and the valence of the touch, were, indeed, rapidly encoded within 160 ms of video onset, reflecting swift early visual processing. Importantly, we also examined for the first time the time course of information processing related to threat and pain in visual touch contexts. Our results showed that these dimensions, along with arousal, were most clearly represented from around 260 ms, primarily involving activity in visual regions. Frequency decoding further revealed that body cues were predominantly encoded in the theta, alpha, and low beta bands (~6–20 Hz), while sensory and emotional-affective features were mainly represented in the delta, theta, and alpha bands (~1–13 Hz). Extending prior work (Lee Masson & Isik, 2023; McMahon & Isik, 2023) beyond social touch, our findings generalize the crucial role of rapid feedforward visual pathways in detecting sensory features and emotional salience during the observation of detailed touch interactions of all kinds.

## 2 Methods

### 2.1 Ethics statement

This study was approved by the Western Sydney University ethics committee (project number: 15644), and informed written consent was obtained from all participants.

### 2.2 Stimuli and video rating procedure

**Fig. 1.**
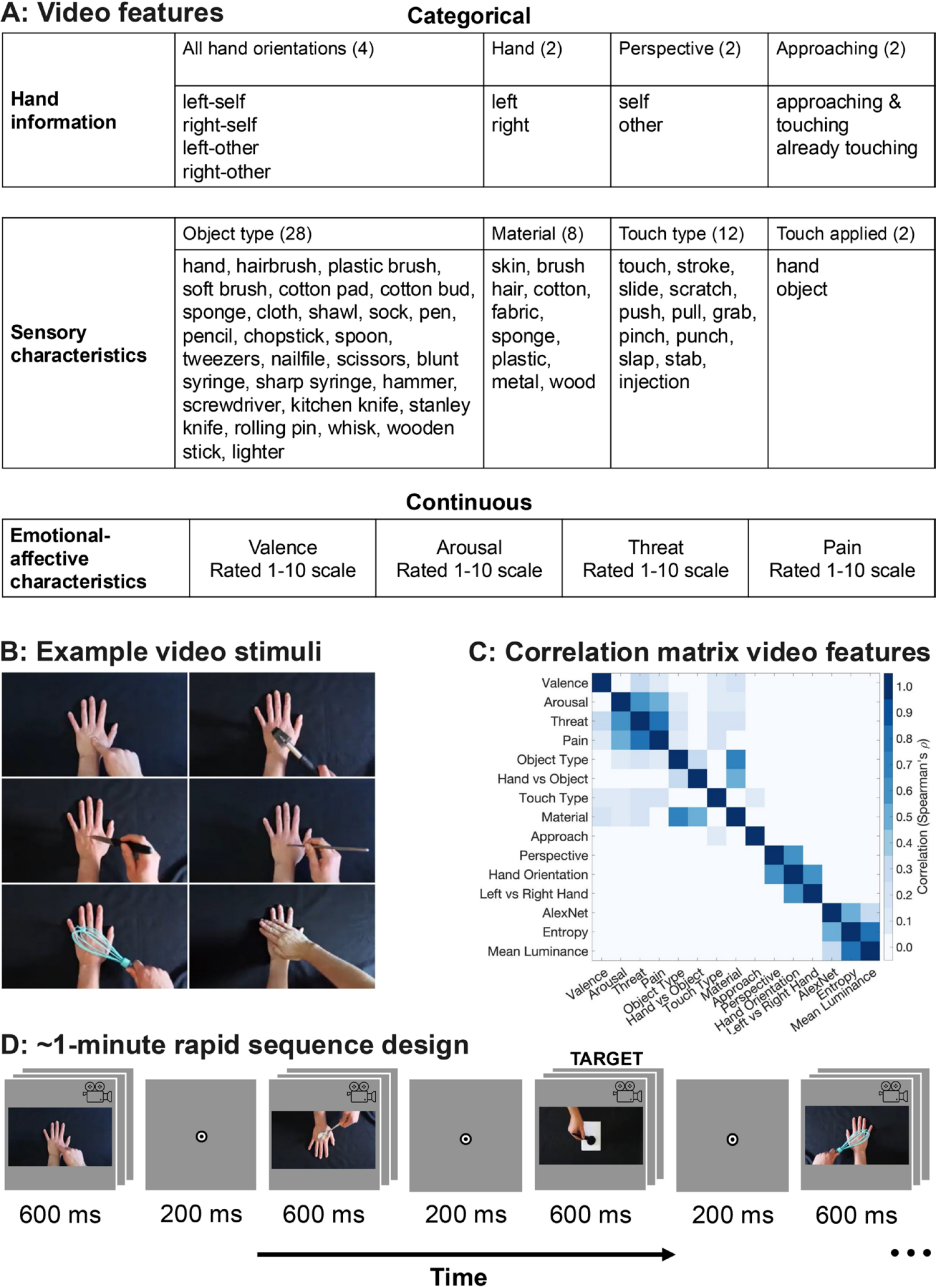
Experimental Stimuli and Design. (A) Table of features used in the regression and decoding analysis, showing the continuous and categorical characteristics extracted from each video. (B) Middle frames from a video subset, presented in four orientations during the experiment to depict left/right hand and self/other perspectives. (C) The overlap of information between the different touch features related to the videos was assessed by creating an RDM for each feature and correlating the models with one another. (D) Example sequence for the EEG experiment. Participants initiated the sequence by pressing the space bar; the 600 ms videos appeared interspersed by 200 ms blank intervals. The task involved counting target trials, on which a white object was touched rather than a hand. Each sequence duration ranged from 54 to 78 s.

We used a set of stimuli previously developed and validated elsewhere (The Validated Touch-Video Database: Smit & Rich, 2025). This original set includes 90 videos of varying lengths that depict various types of tactile interactions with a left hand shown from a first-person perspective (Fig. 1B). The videos differ along several dimensions, including arousal, perceived threat, hedonic qualities (neutral, pleasant, unpleasant, or painful), type of touch (e.g., stroking, pressing, stabbing), and whether the touch involved skin contact with another hand or an object (such as a brush, hammer, or knife). For the current study, we standardized all video lengths to 600 ms, centered on the touch event, comprising 15 frames at a rate of 25 frames per second. The videos measured 256 pixels in width and 144 pixels in height and were presented on a screen positioned approximately 60 cm from the participant (6.2° visual angle). To explore different perspectives, we presented the videos in four orientations achieved by horizontal, vertical, or combined flips, illustrating touch to either the left or right hand from self-oriented or other-oriented viewpoints, totaling 360 stimuli.

To confirm that our modifications (shorter duration, smaller presentation size, and different orientations) did not impact the perceived attributes of the touch compared to the original videos that were previously rated, we recruited a separate cohort of participants to evaluate the modified clips using the same criteria as the original dataset (these participants were independent from the participants in the EEG task). There were 86 participants (72 female, 14 male, mean age 26.3, age range 18–57 years, 76 right-handed, 9 left-handed, and 1 ambidextrous) for the online video rating task. Participants were recruited through Western Sydney University and received course credit or payment. We administered four distinct questionnaires via Qualtrics, each showing the abbreviated 90 videos in only one of the four orientations (e.g., a participant would only see touches to a right hand in a first-person perspective). Each participant completed only one questionnaire, with at least 20 participants contributing to each orientation condition. We included the same four questions from the original database study: (1) How would you categorize the touch in this video? (single answer: neutral, pleasant, unpleasant, painful); (2) How [pleasant/unpleasant/painful] (based on the previous answer) was the touch?; (3) How threatening was the touch?; and (4) How arousing was this video? (arousing in terms of feeling, emotion, or response). The last three questions were rated on a scale from 1 (not at all) to 10 (extremely). The results indicated strong correlations with original ratings (see Supplementary Table 1). We used the arousal, threat, and pain ratings from the original video database in our analyses, as described below. To generate a single valence score for each video, we used the percentage of participants who categorized the video as either pleasant or unpleasant and then applied Principal Component Analysis (PCA) to these percentages. We extracted the first principal component to create a unified valence score.

### 2.3 EEG experiment

We recruited 80 participants for the EEG experiment (54 female, 24 male, 2 non-binary, mean age 30.1 years, age range 18–76 years, 68 right-handed, 9 left-handed, and 3 ambidextrous). We included both left- and right-handed participants, as handedness was not expected to meaningfully impact interpretation of the results. The stimuli depict a model touching either her left or right hand with the opposite hand, ensuring a balanced presentation of both hands. Participants were recruited through Western Sydney University and received course credit or payment. We used a rapid sequence design (e.g., Grootswagers et al., 2019a, 2022) where each participant viewed 32 sequences of 90 videos presented in a randomized but counterbalanced order. To maintain participant attention, additional target stimuli, which involved touch interactions between a hand and a white object, rather than another hand, were interspersed randomly among the normal touch videos and were excluded from the analysis (Fig. 1B). Each sequence contained 1 to 9 target stimuli, with a minimum of 12 non-target stimuli between consecutive targets. Participants were tasked with counting these targets and reporting their count at the end of the sequence via the top row numbers on the keyboard, receiving immediate feedback on their accuracy. Participants achieved an average accuracy of 81.21% (SD = 18.83%) on this target-detection task, demonstrating their high engagement. Each sequence ranged in duration from 54 to 78 s, depending on the number of target trials. The videos, each 600 ms in duration, were separated by a 200 ms inter-video gap and displayed against a light grey background on a 24-inch ViewPixx monitor. Participants were instructed to take breaks as needed between sequences and self-initiated each sequence with a keypress. The EEG task included a total of 2880 non-target trials (each unique video presented eight times) alongside a variable number of target trials, going for approximately 55 min including breaks. The experiment was conducted using Python and PsychoPy software version 2023.3.1 (Peirce et al., 2019). Additionally, we administered four short questionnaires for a separate project, which are not included in the current analyses. These were done at the end of the experiment and required an extra 15 min to complete.

### 2.4 EEG recordings and preprocessing

EEG data were continuously recorded using a 64-channel BioSemi Active-Two electrode system at a sampling rate of 2048 Hz (BioSemi, Amsterdam, The Netherlands). Voltage offsets were maintained below 20 mV. The electrode placement adhered to the 10/20 international standard (Jasper, 1958; Oostenveld & Praamstra, 2001), and offline preprocessing was performed using the Python MNE toolbox version 1.7 (Gramfort et al., 2014). We re-referenced the data to a common average followed by band-pass filtering using a 0.1 Hz high-pass and a 100 Hz low-pass filter to remove slow drifts and high-frequency artifacts. The data were then downsampled to 200 Hz to reduce the data size and computational load. Baseline correction was applied using a window from -100 to 0 ms before stimulus onset, followed by temporal smoothing with a 10-sample (50 ms) moving average filter to reduce short-term fluctuations. Epochs were extracted from -100 to 800 ms relative to each stimulus onset, with no additional preprocessing. Voltages from each channel at every time point were used for subsequent analyses.

### 2.5 Decoding analysis

To analyze the neural encoding of both continuous and categorical aspects of observed visual touch in the EEG data (Fig. 1A), we employed a combination of time-resolved regression and classification analyses (Grootswagers et al., 2017). These analyses were conducted in Python using the MNE toolbox (Gramfort et al., 2014). Continuous aspects, including subjective ratings of valence, arousal, threat, and pain from the Validated Touch-Video Database (Smit & Rich, 2025: rated on a scale from 1 to 10), were predicted using regression. Categorical aspects included all four hand orientations (i.e., hand x perspective), hand (i.e., left vs. right), and perspective (i.e., self vs. other), whether a hand was coming in to touch the other hand or whether the touch was already occurring from the start of the video, object type (e.g., knife, brush), material (e.g., metal, wood; or skin when touched by a hand), touch type (e.g., touch, stroke), and whether the touch was applied by another hand or an object. These categories were all manually coded for this study and decoded using classification. This approach allowed us to assess how accurately the EEG data (channel voltages recorded from 64 electrodes) could predict the subjective ratings taken from the video database (note the videos were rated by a large and separate sample of participants: Smit & Rich, 2025) and distinguish between different categories of touch.

For both the classification and regression analyses, we employed a sliding estimator over time, training and testing on the same time point, in combination with leave-one-sequence-out cross-validation. In each iteration of the cross-validation, we trained our models on 31 sequences and tested on the remaining sequence, repeating this process 32 times. This approach ensured that each sequence was used as a test set once. For the classification and regression, we first standardized all input features by transforming the data to have a mean of zero and a standard deviation of one, preventing features with larger scales from disproportionately influencing the model. For the regression analysis, we used ridge regression with an alpha parameter of 0.5, which helps to control for overfitting by adding a penalty to the model coefficients. The regression model was trained on EEG data to predict continuous emotional dimensions (valence, arousal, threat, and pain) rated on a 1-10 scale for each video in a separate study (Smit & Rich, 2025). Prediction performance was assessed by correlating the model’s cross-validated ratings predicted from the EEG data with the subjective video ratings, with this correlation compared to a chance level of zero. In the classification analysis, we applied regularised Linear Discriminant Analysis (LDA) to classify patterns of neural activity associated with the different categorical features. The performance of the classification model was evaluated using balanced accuracy, which accounts for class imbalances by averaging the accuracy across all classes, ensuring that the classification performance is not biased toward more frequent classes. The balanced accuracy was then compared to a chance level, which was determined based on the number of possible labels (e.g., with 10 labels, the chance level would be 10%).

We conducted an exploratory channel searchlight analysis to examine which EEG sensors contributed most to decoding performance at specific time points. This approach, commonly used to explore spatial patterns in neural decoding (Grootswagers et al., 2019b; Moerel et al., 2024; Robinson et al., 2019; Smit et al., 2023), involves performing decoding within local clusters of neighboring electrodes across the scalp. For each time point of interest, we defined a cluster of electrodes centered on a given EEG channel and including its nearest neighbors. We applied the same decoding analysis described above within each of these spatial clusters using a regularized LDA classifier for categorical classification and ridge regression for continuous features. The decoding results were stored at the central channel of each cluster, resulting in a time-by-channel accuracy or correlation map for each participant. This method enables spatially resolved decoding, allowing us to identify which sensors contributed most to the neural discrimination of each feature. We created topographies for nine time points, ranging from -50 ms to 750 ms with 100 ms intervals.

To characterize the spectral features underlying visual touch encoding, we extended our multivariate decoding approach to the frequency domain. For this, the EEG data were kept at 2048 Hz, and we computed the power spectral density for each 800 ms epoch using a Fast Fourier Transform. This process yielded 41 frequency bins spanning 0–50 Hz with an intrinsic resolution of 1.25 Hz per bin. We discarded the 0 Hz bin, which reflects constant (non-oscillatory) signal power, and retained 40 frequency bins (1.25–50 Hz). Decoding was then performed separately at each frequency using the same multivariate regression and classification methods employed in the time-resolved analyses.

To account for potential confounding visual effects, we controlled for key visual characteristics by extracting and regressing out three distinct visual features from the EEG data: entropy, mean luminance, and AlexNet features. Entropy and mean luminance were estimated from the first frame of each video. Entropy was calculated as a measure of the randomness or complexity within the grayscale image, reflecting the variability in pixel intensities. Mean luminance was computed as the average brightness of the pixels in the colored image, providing an effective measure of the overall lightness of the frame. The choice of the first frame is justified by its role in setting the visual tone and brightness for the video. For the AlexNet features, we used the colored middle frame and extracted features from the final convolutional stage (relu5). This stage captures a broad range of visual structure from low-level edges to mid-level shape and texture representations (Cichy et al., 2016). Relu5 can be regarded as a compressed summary of earlier convolutional stages, and among these visual layers, it explained the most variance in our EEG data. The middle frame was selected to capture the typical content of the video, as it likely reflects the central theme or key moment of the interaction, making it a suitable choice for extracting deep neural network features. We then converted the output of relu5 into a one-dimensional feature vector using PCA. The three visual models were created in MATLAB, and the results were loaded into Python for subsequent analyses. Entropy and mean luminance were calculated using standard image processing libraries, while the AlexNet features were derived by applying a pre-trained deep learning model (Krizhevsky et al., 2012). Each of these visual features (entropy, mean luminance, and the AlexNet PCA component) was treated as a separate model in our regression analysis. We regressed out these features from the EEG signal by fitting a linear regression model at each channel and time point within each fold of the cross-validation process. The residuals from this regression were then used for fitting the classification and regression models, ensuring that the EEG data reflected neural responses to touch perception rather than variance in low-level visual input.

To assess the association between various features in our decoding analysis, we used dissimilarity matrices (Kriegeskorte & Kievit, 2013). These were generated from perceptual ratings from the Validated Touch-Video Database (Smit & Rich, 2025) for continuous features, manual coding results for categorical features, and results from the visual models AlexNet, entropy, and mean luminance. Dissimilarity matrices are particularly useful as they allow for the comparison of both continuous and categorical data on a common scale, facilitating a comprehensive analysis across different data types (Kriegeskorte & Kievit, 2013). We employed Spearman’s rank correlation to evaluate the relationships among these models, uncovering how different characteristics correlate with one another (Fig. 1C).

### 2.6 Statistical inference

We used Bayes factors to evaluate the evidence for both the null (chance decoding) and alternative (above-chance decoding) hypotheses (Dienes, 2011; Kass & Raftery, 1995; Morey et al., 2016; Rouder et al., 2009). Bayes factors were computed using the ‘BayesFactor’ package from R (Morey et al., 2018), integrated into Python via the rpy2 interface. We applied a half-Cauchy prior for the alternative hypothesis to capture above-chance effects. The prior was centered around chance with the default width r = 0.707 (Jeffreys, 1998; Rouder et al., 2009; Wetzels et al., 2011). Based on previous recommendations (Teichmann et al., 2022), we omitted the interval from d = 0 to d = 0.5 from the prior to disregard effect sizes considered too small to be relevant. For the topographical plots and the frequency analysis, we did not apply a null interval as we expected smaller effect sizes due to fewer sensors and small frequency bins capturing less information. Bayes factors quantify the strength of evidence, with a Bayes factor greater than 1 indicating support for the alternative hypothesis, and less than 1 supporting the null (Jeffreys, 1998). A Bayes factor of 6, for example, suggests that the evidence favoring the alternative hypothesis is six times stronger than that for the null, although we avoid strict cut-offs to maintain a focus on the evidence continuum rather than binary outcomes (Dienes, 2011; Kass & Raftery, 1995; Morey et al., 2016; Rouder et al., 2009).

## 3 Results

### 3.1 Time-resolved decoding

We explored the temporal dynamics of neural responses to visually perceived touch. We recorded EEG data while participants watched brief and detailed touch interaction videos, adapted from the Validated Touch-Video Database (Smit & Rich, 2025). This study aimed to address a gap in our knowledge of visual touch perception by examining how the brain processes visual body cues, such as viewing perspective, alongside more complex sensory and emotional-affective interpretations of touch.

**Fig. 2.**
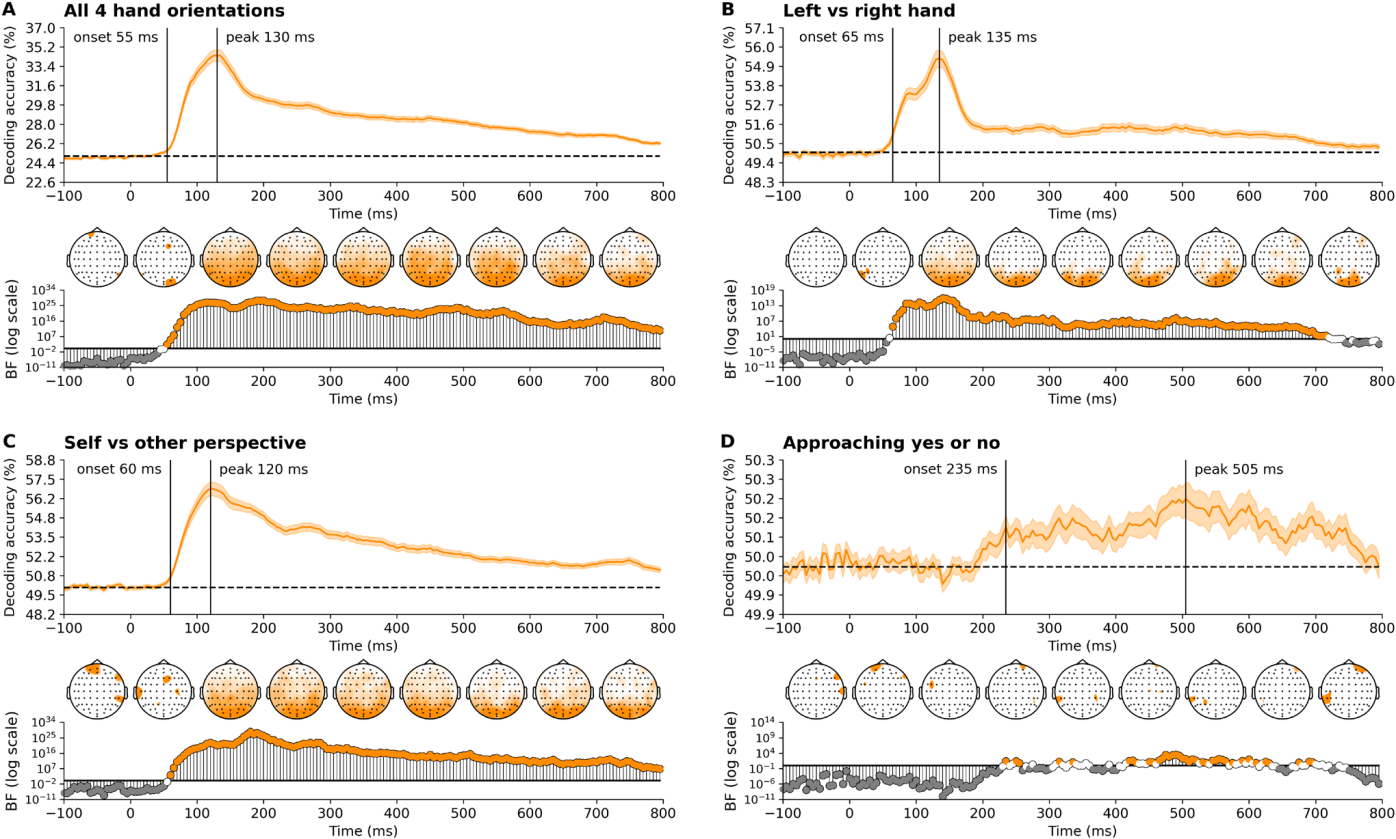
Time course of decoding accuracies of hand information show rapid discrimination between hand orientations and perspectives, with a notably later response for approaching movements. These plots illustrate the time-varying decoding accuracies for (A) all four hand orientations, (B) left versus right hand, (C) self versus other perspective, and (D) the approach of one hand towards another versus an already touching hand. Stimulus onset is at 0 ms. Theoretical chance levels are set at 25% for the four hand orientations and 50% for binary distinctions (e.g., left vs. right hand, self vs. other perspective, approaching yes or no), marked by the horizontal dotted lines. Shaded areas around the plot lines represent the standard error of the mean across participants (N = 80). Below the plots, Bayes factors are displayed on a logarithmic scale. Bayes factors below 1/6 (shown in grey) indicate strong evidence for the null hypothesis, those above 6 (shown in color) indicate strong evidence for the alternative hypothesis, and those between 1/6 and 6 are shown in white. Vertical lines indicate the onset and peak time points of sustained above-chance decoding. Additionally, time-varying topographies derived from the channel-searchlight analysis are presented in color, showing Bayes factors ≥ 3 for individual time points ranging from -50 ms to 750 ms at 100 ms intervals.

We decoded categorical features related to the visually observed touch at each time point to determine when this information appears in the EEG responses. We first focused on the viewing perspective and movement dynamics (Fig. 2). Our findings reveal that hand orientation was decodable very early, starting at approximately 60 ms and peaking around 120-130 ms, including distinctions of hand (left vs. right) and perspective (self vs. other). A distributed pattern of channels across the scalp contributed to the decoding of these aspects. Evidence for above-chance decoding for the more intricate interaction dynamics, specifically, whether one hand was approaching the other to touch or if the touch was already occurring from the first frame, was present from about 230 ms, peaking later around 500 ms. These results indicate that visual information about the body part touched, and the likely recipient (self or other), is processed rapidly via the visual pathway.

**Fig. 3.**
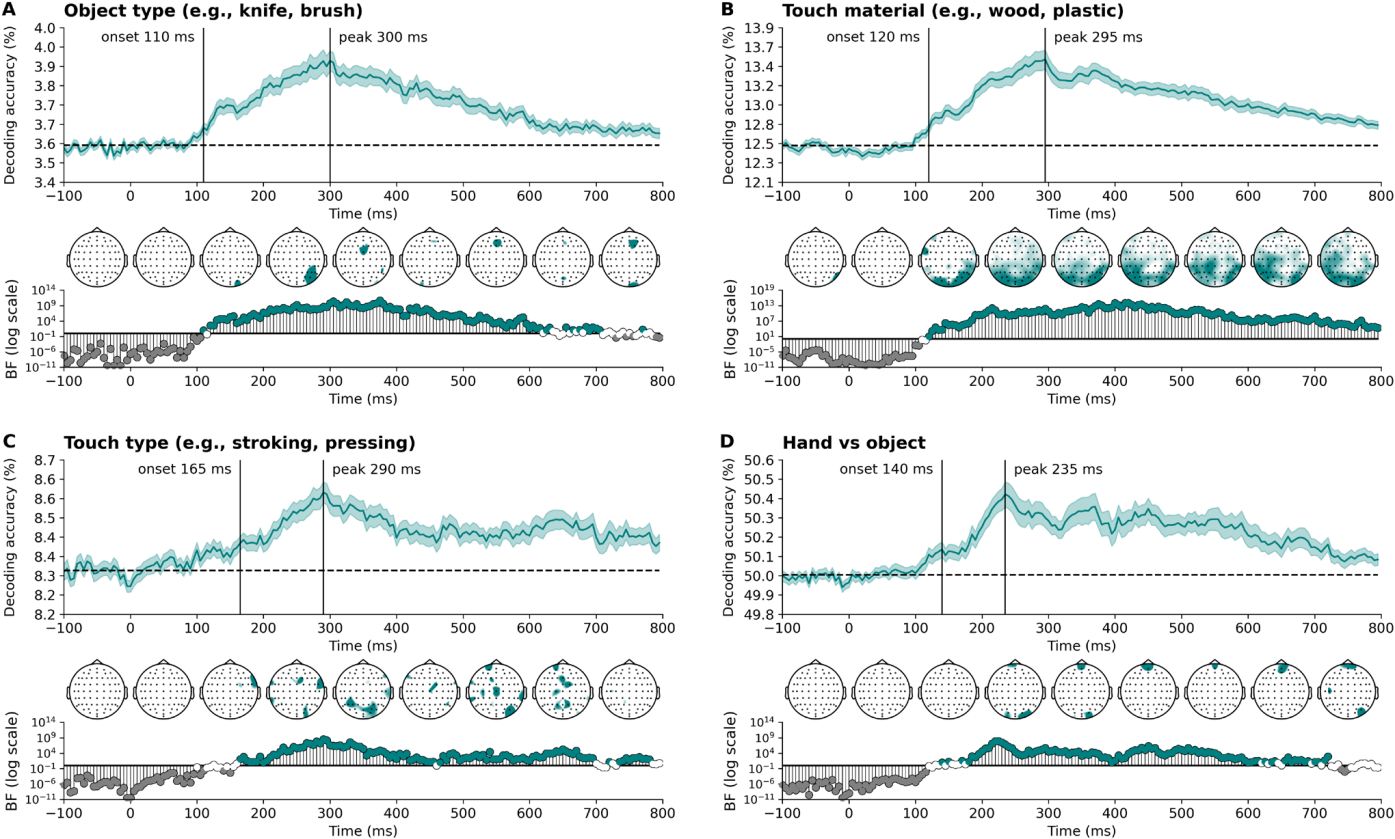
Time course of decoding accuracies of sensory characteristics show rapid discrimination of object, material, touch type, and whether the touch was applied by a hand or an object. These plots illustrate the time-varying decoding accuracies for (A) object type, (B) the material involved in the touch (note that contact between hands is included as a label), (C) touch type, and (D) touch applied by a hand vs. an object. Stimulus onset is at 0 ms. Theoretical chance levels are set at 3.6% for object type, 12.5% for material, 8.3% for touch type, and 50% for hand vs. object, marked by the horizontal dotted lines. Shaded areas around the plot lines represent the standard error of the mean across participants (N = 80). Below the plots, Bayes factors are displayed on a logarithmic scale. Bayes factors below 1/6 (shown in grey) indicate strong evidence for the null hypothesis, those above 6 (shown in colour) indicate strong evidence for the alternative hypothesis, and those between 1/6 and 6 are shown in white. Vertical lines indicate the onset and peak time points of sustained above-chance decoding. Additionally, time-varying topographies derived from the channel-searchlight analysis are presented in colour, showing Bayes factors ≥ 3 for individual time points ranging from -50 ms to 750 ms at 100 ms intervals.

Next, we examined the sensory characteristics of the visually observed touch (Fig. 3). The type of object and related material attributes, which varied in texture and other properties (e.g., metal may be perceived as cold and a cloth as warm), were both decoded rapidly within 110–120 ms. The ability to distinguish between a touch applied by a hand, involving direct skin contact, or with an object, emerged slightly later, by about 140 ms. The type of touch (e.g., stroking versus pressing) was subsequently decodable by 165 ms. Information about these sensory aspects was sustained for some time, with peaks occurring around 230–300 ms, indicating when most information was present. Processing of these sensory aspects first appeared at posterior (visual) electrode sites, then shifted to more central and finally frontal/temporal electrodes over time, particularly for the material involved in the touch. These findings highlight representations of sensory information being present during the initial stages of visual processing.

**Fig. 4.**
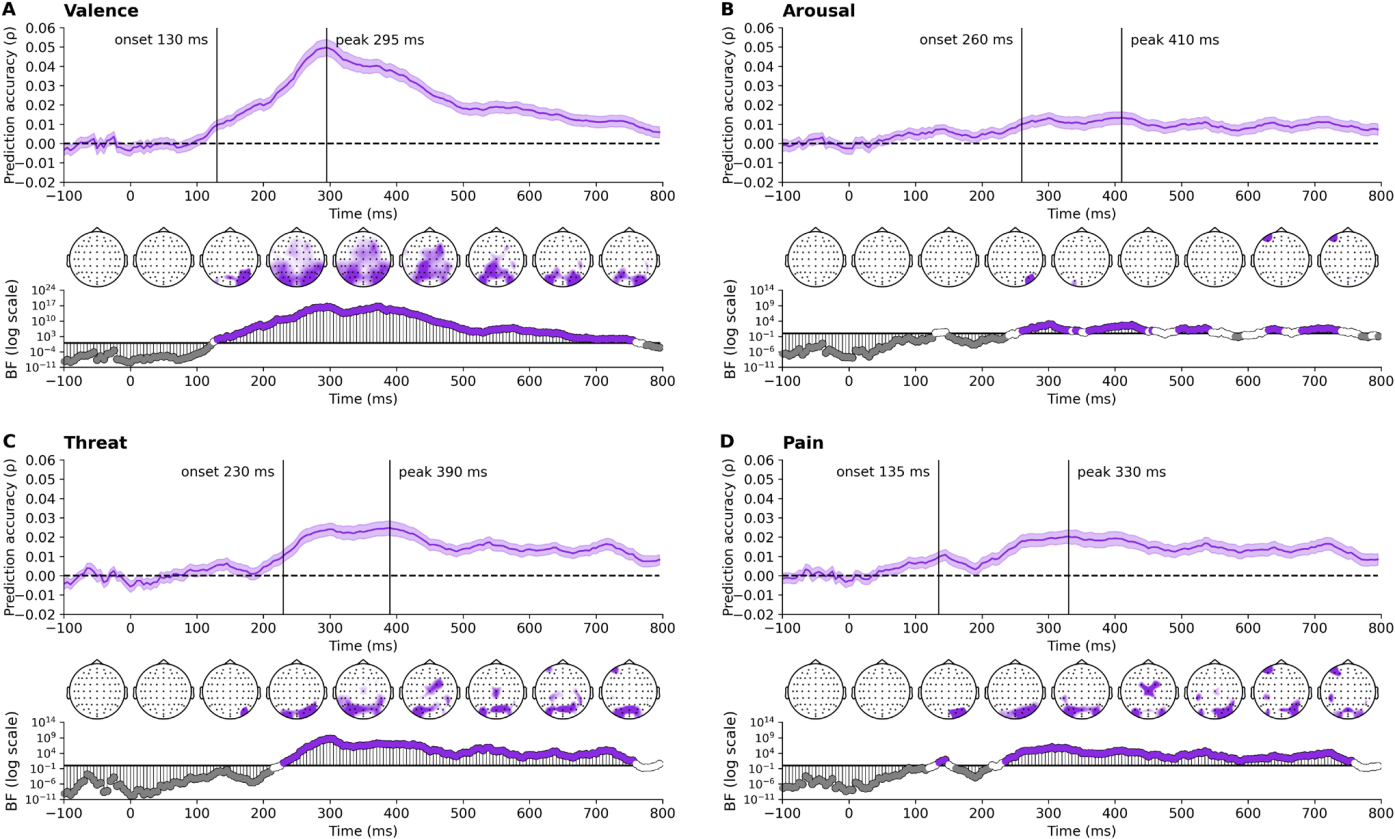
Time course of decoding accuracies of emotional-affective characteristics show rapid discrimination of valence, with a slightly later response for more complex evaluations of arousal, threat and pain. These plots illustrate the prediction accuracy for (A) valence, (B) arousal, (C) threat, and (D) pain. Stimulus onset is at 0 ms. Theoretical chance levels are set at 0, marked by the horizontal dotted lines. Shaded areas around the plot lines represent the standard error of the mean across participants (N = 80). Below the plots, Bayes factors are displayed on a logarithmic scale. Bayes factors below 1/6 (shown in grey) indicate strong evidence for the null hypothesis, those above 6 (shown in color) indicate strong evidence for the alternative hypothesis, and those between 1/6 and 6 are shown in white. Vertical lines indicate the onset and peak time points of sustained above-chance decoding. Additionally, time-varying topographies derived from the channel-searchlight analysis are presented in color, showing Bayes factors ≥ 3 for individual time points ranging from -50 ms to 750 ms at 100 ms intervals.

Next, we investigated the emotional-affective aspects of the visually observed touch (Fig. 4). Information about touch valence was clearly present in the neural data early on, from approximately 130 ms. This was supported by initial decoding at posterior (visual) electrode sites, which progressively shifted to more central and then frontal scalp locations over time. Information about the level of pain was briefly present in the data at a similar time, from approximately 135 ms; however, evidence supports clearest decoding from approximately 240 ms. Information related to threat and arousal was decodable from about 230 ms and 260 ms respectively, indicating a more delayed response that may involve higher cognitive integration to evaluate potential danger. All dimensions peaked around 300-400 ms. Though the decoding of pain, threat, and arousal occurred somewhat later in time compared to valence, the spatial decoding patterns still suggest strong involvement from visual regions. These findings illustrate that while potentially more basic emotional responses to touch, such as valence, are processed quickly, and via the initial visual processing pathway, more complex evaluations about potential harm involve slightly longer neural processing times and potentially deeper cognitive involvement.

### 3.2 Frequency-domain decoding

**Fig. 5.**
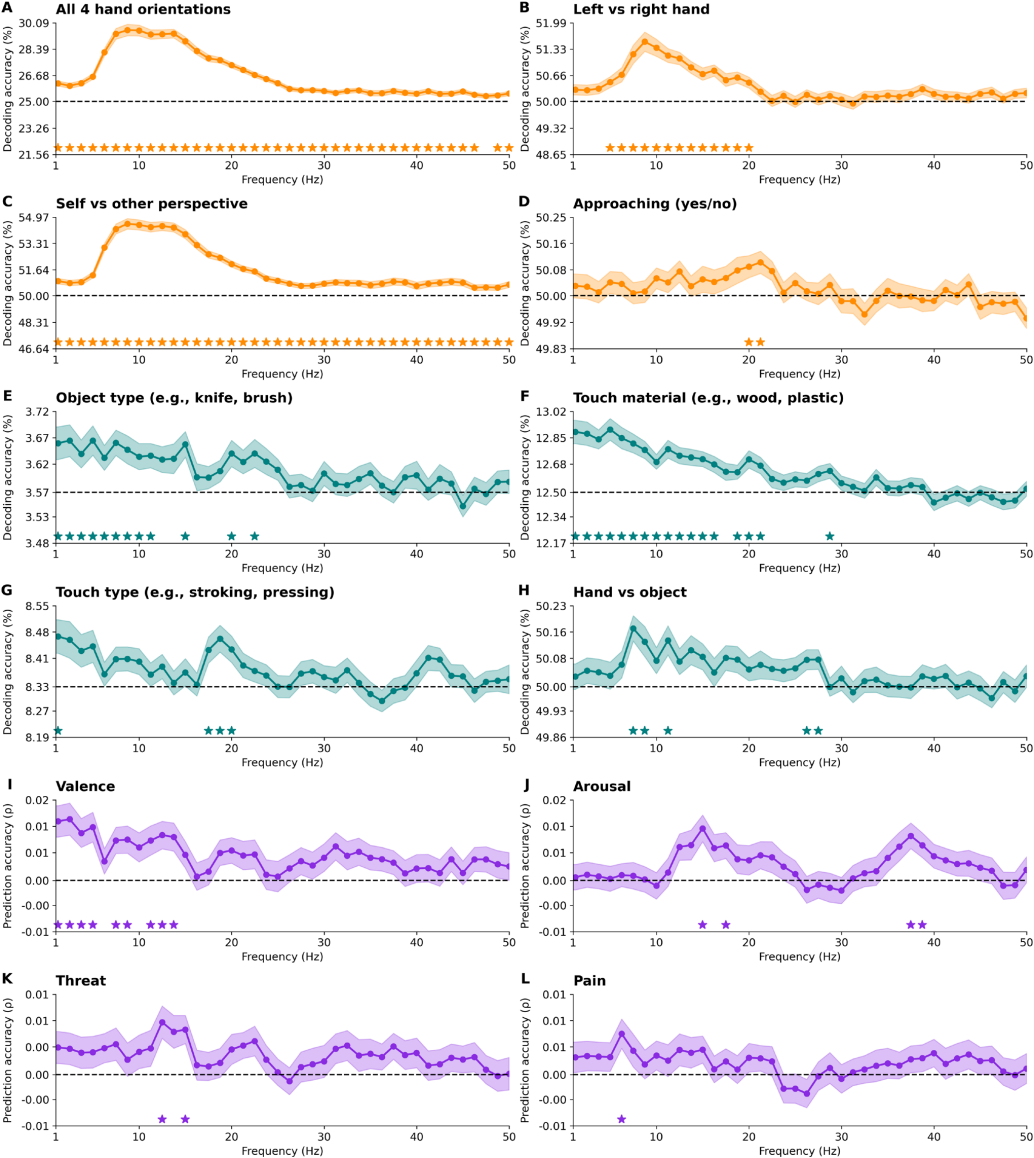
Frequency‐domain decoding. These plots illustrate the performance of frequency-domain decoding across a spectrum of 1 to 50 Hz for (A) all four hand orientations, (B) left versus right hand, (C) self versus other perspective, (D) the approach of one hand toward another versus an already touching hand, (E) object type, (F) the material involved in the touch, (G) touch type, (H) touch applied by a hand vs. an object, (I) valence, (J) arousal, (K) threat, and (L) pain. Theoretical chance levels are marked by the horizontal dotted lines. Shaded areas around the plot lines represent the standard error of the mean across participants (N = 80). Below the plots, Bayes factors above 6 are displayed as stars.

The frequency domain analysis revealed distinct oscillatory signatures for the various features (Fig. 5). Body cues (hand orientation, self vs. other perspective, left vs. right hand) were decodable over a broad frequency range, peaking in the theta, alpha, and low beta bands (∼6–20 Hz), and remaining above chance into the low gamma range (up to 50 Hz). Decoding of whether a hand was approaching in the videos or already touching from the start was most prominent in the beta band (∼20–21 Hz). For sensory features, object type and material were best decoded in the delta, theta, alpha, and beta bands (∼1–18 Hz), with some additional contributions in the low gamma range (up to ∼28 Hz). Touch type was decodable in the delta band (∼1 Hz) and again in the low gamma range (∼18–20 Hz). Whether the touch involved direct skin contact or another object was detectable in the alpha band (∼8–13 Hz), and again in the beta range (∼26–27 Hz). Emotional-affective features (valence, arousal, threat, and pain) were overall weaker, but still showed frequency decoding above chance. Valence was clearly reflected in the delta, theta, and alpha bands (∼1–13 Hz). Arousal, threat, and pain showed more limited decoding, with contributions in the theta range (∼6 Hz) for pain, the low beta range for threat (∼12–15 Hz), and both low beta and gamma frequencies for arousal (∼15–17 Hz and ∼38 Hz). Altogether, these results indicate that body cues are encoded across a broad spectral range, especially within the theta, alpha, and low beta bands, while sensory and emotional-affective features are primarily represented in the delta, theta, and alpha bands, with some contributions in the higher beta and gamma bands.

## 4 Discussion

This study is the first to use EEG to investigate the neural dynamics of visually perceived touch across the dimensions of body cues, sensory qualities, and emotional-affective features, and to do so in a large sample of observers (N = 80). Participants viewed 90 close-up video clips of hand-based touch interactions, adapted from the Validated Touch-Video Database (Smit & Rich, 2025), presented as touch to either the left or right hand and from a self- or other-perspective. By carefully controlling for low-level visual properties, we ensured that the observed effects reflected meaningful touch-related information rather than structural stimulus differences. Our multivariate decoding analyses revealed that most features were rapidly represented during early visual processing, highlighting the strong contribution of visual pathways to touch perception.

Our finding that valence information is encoded during early visual processing (as early as 130 ms, peaking around 300 ms) parallels prior work by Lee Masson and Isik (2023) showing that emotional-affective aspects of social touch are processed within a similar timeframe (~150–180 ms). However, unlike that study, which used rich social contexts (e.g., people hugging), we used touch stimuli showing one hand touching another (from the same person), allowing us to isolate sensory and emotional encoding without broader social cues. We note, however, that Lee Masson and Isik (2023) reported valence and arousal information arising nearly simultaneously, and that valence could not uniquely explain variance in their EEG signal once effects of other features (sociality and arousal) were accounted for, suggesting some variability across paradigms. Nevertheless, this early availability of emotional-affective information across different contexts highlights the critical role of visual pathways in detecting emotional salience, with visually selective areas engaged before somatosensory regions (Lee Masson & Isik, 2023). Our findings align with broader evidence that the perception of complex social-emotional information when observing social interactions (McMahon & Isik, 2023) and emotional images (Grootswagers et al., 2020) is driven largely by automatic, bottom-up visual processes. These mechanisms enable the visual system to support rapid, adaptive responses to observed events, underscoring its key role in interpreting complex touch interactions.

Other affective dimensions emerged somewhat later in time. Pain-related information was briefly present around 135 ms, followed by more sustained decoding from 240 ms, peaking at 330 ms. This time course aligns with prior research on visual depictions of hands in painful scenarios, where event-related potential (ERP) modulations differentiated painful from neutral stimuli at ~140 ms and ~380 ms, with early amplitudes (140–180 ms) correlating with subjective ratings of observed pain and discomfort (Fan & Han, 2008). Our findings also align with recent immersive virtual reality research using ERP and time-frequency analyses, which demonstrated early visual discrimination of pleasant versus painful touch (160–400 ms) and modulation by viewing perspective (190–310 ms) when participants observed touch to an embodied avatar (Nicolardi et al., 2025). These results complement our decoding-based approach and point to future work testing how emotional-affective processing interacts with visual perspective.

Although prior studies have mapped the time course of threat perception in other contexts, such as facial expressions (showing modulations between 120–280 ms and later around 400–500 ms; Schupp et al., 2004; Williams et al., 2006), little is known about its temporal dynamics during visually observed touch. In our study, threat-related information emerged most clearly around 230 ms, peaking at 390 ms. Similarly, arousal-related information appeared later, from 260 ms, peaking at 410 ms. These later dynamics may reflect greater cognitive effort to assess potential danger. Decoding onsets may also to some extent be shaped by stimulus dynamics. Some features (e.g., hand orientation, object type) are readily available from the very first frame, while others may become more salient with motion or context across several frames. Thus, the later decoding of threat, pain, and arousal may indicate both evaluative processes and the temporal availability of diagnostic visual information. Here, we do not aim to make precise claims about temporal dissociations between dimensions. Rather, our results suggest that most features are represented early within the initial feedforward sweep, while others (particularly threat and arousal) require relatively more time before they are clearly decodable. Despite the delayed timing, the continued involvement of posterior electrode sites suggests that both immediate and more complex emotional responses to touch are integrated within the visual processing system.

Analysing spatial decoding patterns over time, we found that the decoding of sensory and emotional aspects, especially material properties and valence, began over posterior (visual) electrodes and quickly extended to central and frontal scalp regions. This posterior-to-central progression may reflect the simulation of sensory properties in visually observed touch, potentially engaging the observer’s somatosensory system (Gallese et al., 2004; Keysers & Gazzola, 2009; Lee Masson & Isik, 2023; Peled-Avron & Woolley, 2022). For material properties, decoding patterns also progressed toward temporal electrode sites. The brain’s assessment of materials presumably integrates both physical properties and social-emotional meaning, for example the comfort of soft fabrics versus the discomfort of rough textures or the intimacy of skin-to-skin versus object-to-skin contact. Processing these dimensions would likely involve temporal regions linked to body posture and emotional content extraction, including the extrastriate body area, fusiform body area, and posterior superior temporal sulcus (Keysers & Gazzola, 2006; Peelen et al., 2007; Peelen & Downing, 2007), as well as regions critical for object property recognition, such as the lateral occipital complex and fusiform gyrus (Kanwisher et al., 1996; Malach et al., 1995). While prior research on focused touch interactions has emphasized the observer’s somatosensory cortex in producing vicarious sensory experiences (Adler & Gillmeister, 2019; Adler et al., 2016; Bufalari et al., 2007; Galilee & McCleery, 2016; Martínez-Jauand et al., 2012; Pihko et al., 2010; Rigato et al., 2019a, 2019b; Smit et al., 2023; Streltsova and McCleery, 2014), our time-course findings highlight a critical role for early visual processing in rapidly extracting touch-related information, which then appears to extend to more central, temporal, and frontal scalp regions.

In addition to exploring spatial-temporal dynamics, we investigated how different frequencies contribute to the neural encoding of observed touch. This approach revealed distinct spectral signatures for different types of information: body-related cues were decodable across a broad frequency range, with strongest contributions in the theta, alpha, and low beta bands, while sensory and emotional-affective features, such as object type, material, valence, threat, and pain, were primarily reflected in the delta, theta, and alpha ranges. These findings align with prior work linking low-frequency oscillations to high-level cognitive processes and integrative perceptual functions (Başar et al., 2001; Harmony, 2013; Knyazev, 2007). Delta and theta activity, in particular, has been associated with emotion-related visual processing (Güntekin & Başar, 2014; Knyazev, 2007, 2012) and with the vicarious processing of affectionate touch (Schirmer & McGlone, 2019). Our results suggest that sensory and affective evaluations of touch, such as detecting whether it is pleasant or unpleasant, engage similar low-frequency dynamics. In addition, suppression of alpha/mu activity over somatosensory regions has been linked to vicarious sensory experiences (Peled-Avron et al., 2016; Whitmarsh et al., 2011; Yang et al., 2009), while alpha activity has also been associated with attention to tactile events experienced directly on one’s own body (Whitmarsh et al., 2017). Emerging evidence further suggests that perceptual content during visual imagery is preferentially represented in the alpha band (Arnold et al., 2024; Stecher & Kaiser, 2024; Xie et al., 2020). In line with these prior findings, our results similarly show that information related to visually perceived touch is primarily processed in lower-frequency bands, possibly reflecting neural mechanisms underlying tactile attention, imagery, and self–other differentiation.

We note that some decoding accuracies in our study were modest; however, this does not imply the absence of meaningful neural information. Low accuracy values can still reflect robust and theoretically significant representations, particularly when supported by clear temporal dynamics and strong statistical evidence—decoding accuracy should not be conflated with effect size (Combrisson & Jerbi, 2015; Hebart & Baker, 2018). Our interpretive decoding approach focused on identifying when the brain distinguishes between touch dimensions, rather than maximizing predictive accuracy. The use of minimal preprocessing and tightly controlled stimuli, though beneficial for reducing confounds, likely produced somewhat low signal-to-noise ratios (Grootswagers et al., 2017). Nonetheless, the consistent temporal patterns and Bayesian evidence highlight meaningful neural encoding across all aspects of observed touch.

Future research could explore how individual differences, such as empathy and vicarious touch experiences, shape the neural encoding of visually perceived touch. For example, individuals who report physically feeling sensations when observing others being touched (Gillmeister et al., 2017; Smit et al., 2025a) may process observed touch differently from those without such experiences. These heightened vicarious responders might show distinct temporal patterns when encoding tactile information (Smit et al., 2023), potentially with reduced differentiation between self and other perspectives due to diminished self–other boundaries (Banissy et al., 2009; Ward & Banissy, 2015). In addition to trait-level factors, future work could investigate how moment-to-moment, state-level appraisals, such as perceived threat or pain in response to specific stimuli, modulate neural responses. While the present study used validated ratings from an independent sample to minimize task-related biases, incorporating real-time subjective measures could offer valuable insights into individual variability in affective touch processing. Finally, future research might examine the neural dynamics of purely sensory touch interactions devoid of any social context. Although our study focused on close-up touches to isolate detailed tactile features, the presence of two interacting hands still introduced an inherent social element. Removing this factor, for example by using mechanical touch stimuli, could help disentangle the neural mechanisms of sensory processing from those shaped by social context.

In conclusion, our findings reveal the significant role rapid and automatic visual processing plays in extracting the sensory and emotional characteristics of perceived touch. This has broader implications for developing more effective and nuanced models of sensory processing, and for applications in areas that focus on multisensory integration, such as neuroprosthetics where visual feedback could enhance the perception of touch.

## Supplementary Material

Supplementary Material

## Data Availability

Stimulus presentation and analysis scripts are available on the Open Science Framework (OSF: https://osf.io/ntfae/). Raw EEG and behavioral data are provided in BIDS format via OpenNeuro (https://openneuro.org/datasets/ds005662; Smit et al., 2025b). Both the adapted videos used in this project and the original videos along with their validation data are available on OSF (https://osf.io/jvkqa/).

## References

[IMAG.a.1017-b1] Addabbo, M., Bolognini, N., & Turati, C. (2021). Neural time course of pain observation in infancy. Developmental Science, 24, e13074. 10.1111/desc.13074 33314507

[IMAG.a.1017-b2] Addabbo, M., Quadrelli, E., Bolognini, N., Nava, E., & Turati, C. (2020). Mirror-touch experiences in the infant brain. Social Neuroscience, 15, 641–649. 10.1080/17470919.2020.1840431 33084498

[IMAG.a.1017-b3] Adler, J., & Gillmeister, H. (2019). Bodily self-relatedness in vicarious touch is reflected at early cortical processing stages. Psychophysiology, 56, e13465. 10.1111/psyp.13465 31464351

[IMAG.a.1017-b4] Adler, J., Schabinger, N., Michal, M., Beutel, M. E., & Gillmeister, H. (2016). Is that me in the mirror? Depersonalisation modulates tactile mirroring mechanisms. Neuropsychologia, 85, 148–158. 10.1016/j.neuropsychologia.2016.03.009 26970140

[IMAG.a.1017-b5] Arnold, D. H., Saurels, B. W., Anderson, N., Andresen, I., & Schwarzkopf, D. S. (2024). Predicting the subjective intensity of imagined experiences from electrophysiological measures of oscillatory brain activity. Scientific Reports, 14, 836. 10.1038/s41598-023-50760-7 38191506 PMC10774351

[IMAG.a.1017-b6] Banissy, M. J., Kadosh, R. C., Maus, G. W., Walsh, V., & Ward, J. (2009). Prevalence, characteristics and a neurocognitive model of mirror-touch synaesthesia. Experimental Brain Research, 198, 261–272. 10.1007/s00221-009-1810-9 19412699

[IMAG.a.1017-b7] Başar, E., Başar-Eroglu, C., Karakaş, S., & Schürmann, M. (2001). Gamma, alpha, delta, and theta oscillations govern cognitive processes. International Journal of Psychophysiology, 39, 241–248. 10.1016/S0167-8760(00)00145-8 11163901

[IMAG.a.1017-b8] Boehme, R., Hauser, S., Gerling, G. J., Heilig, M., & Olausson, H. (2019). Distinction of self-produced touch and social touch at cortical and spinal cord levels. Proceedings of the National Academy of Sciences of the United States of America, 116, 2290–2299. 10.1073/pnas.1816278116 30670645 PMC6369791

[IMAG.a.1017-b9] Bufalari, I., Aprile, T., Avenanti, A., Di Russo, F., & Aglioti, S. M. (2007). Empathy for pain and touch in the human somatosensory cortex. Cerebral Cortex, 17, 2553–2561. 10.1093/cercor/bhl161 17205974

[IMAG.a.1017-b10] Bufalari, I., & Ionta, S. (2013). The social and personality neuroscience of empathy for pain and touch. Frontiers in Human Neuroscience, 7, 393. 10.3389/fnhum.2013.00393 23898249 PMC3724165

[IMAG.a.1017-b11] Butti, N., Urgesi, C., McGlone, F. P., Oldrati, V., Montirosso, R., & Cazzato, V. (2024). To touch or to be touched? Comparing appraisal of vicarious execution and reception of interpersonal touch. PLoS One, 19, e0293164. 10.1371/journal.pone.0293164 38758835 PMC11101113

[IMAG.a.1017-b12] Cichy, R. M., Khosla, A., Pantazis, D., Torralba, A., & Oliva, A. (2016). Comparison of deep neural networks to spatio-temporal cortical dynamics of human visual object recognition reveals hierarchical correspondence. Scientific Reports, 6, 27755. 10.1038/srep27755 27282108 PMC4901271

[IMAG.a.1017-b13] Combrisson, E., & Jerbi, K. (2015). Exceeding chance level by chance: The caveat of theoretical chance levels in brain signal classification and statistical assessment of decoding accuracy. Journal of Neuroscience Methods, 250, 126–136. 10.1016/j.jneumeth.2015.01.010 25596422

[IMAG.a.1017-b14] de Vignemont, F. (2017). Mirror-touch synaesthesia: Intersubjective or intermodal fusion? In Deroy, O. (Ed.), Sensory blending: On synaesthesia and related phenomena (pp. 275–291). Oxford University Press. 10.1093/oso/9780199688289.003.0014

[IMAG.a.1017-b15] Dienes, Z. (2011). Bayesian versus orthodox statistics: Which side are you on? Perspectives on Psychological Science, 6, 274–290. 10.1177/1745691611406920 26168518

[IMAG.a.1017-b16] Fan, Y., & Han, S. (2008). Temporal dynamic of neural mechanisms involved in empathy for pain: An event-related brain potential study. Neuropsychologia, 46, 160–173. 10.1016/j.neuropsychologia.2007.07.023 17825852

[IMAG.a.1017-b17] Galilee, A., & McCleery, J. P. (2016). Neural mechanisms of the observation of human and non-human object touch in children: An event-related potential study. British Journal of Developmental Psychology, 34, 86–100. 10.1111/bjdp.12119 26659431

[IMAG.a.1017-b18] Gallace, A., & Spence, C. (2010). The science of interpersonal touch: An overview. Neuroscience & Biobehavioral Reviews, 34, 246–259. 10.1016/j.neubiorev.2008.10.004 18992276

[IMAG.a.1017-b19] Gallese, V., & Goldman, A. (1998). Mirror neurons and the simulation theory of mind-reading. Trends in Cognitive Sciences, 2, 493–501. 10.1016/S1364-6613(98)01262-5 21227300

[IMAG.a.1017-b20] Gallese, V., Keysers, C., & Rizzolatti, G. (2004). A unifying view of the basis of social cognition. Trends in Cognitive Sciences, 8, 396–403. 10.1016/j.tics.2004.07.002 15350240

[IMAG.a.1017-b21] Gazzola, V., Spezio, M. L., Etzel, J. A., Castelli, F., Adolphs, R., & Keysers, C. (2012). Primary somatosensory cortex discriminates affective significance in social touch. Proceedings of the National Academy of Sciences, 109, E1657–E1666. 10.1073/pnas.1113211109 PMC3382530 22665808

[IMAG.a.1017-b22] Gillmeister, H., Bowling, N., Rigato, S., & Banissy, M. J. (2017). Inter-individual differences in vicarious tactile perception: A view across the lifespan in typical and atypical populations. Multisensory Research, 30, 485–508. 10.1163/22134808-00002543 31287084

[IMAG.a.1017-b23] Gramfort, A., Luessi, M., Larson, E., Engemann, D. A., Strohmeier, D., Brodbeck, C., Parkkonen, L., & Hämäläinen, M. S. (2014). MNE software for processing MEG and EEG data. Neuroimage, 86, 446–460. 10.1016/j.neuroimage.2013.10.027 24161808 PMC3930851

[IMAG.a.1017-b24] Grootswagers, T., Kennedy, B. L., Most, S. B., & Carlson, T. A. (2020). Neural signatures of dynamic emotion constructs in the human brain. Neuropsychologia, 145, 106535. 10.1016/j.neuropsychologia.2017.10.016 29037506 PMC5899060

[IMAG.a.1017-b25] Grootswagers, T., Robinson, A. K., & Carlson, T. A. (2019a). The representational dynamics of visual objects in rapid serial visual processing streams. NeuroImage, 188, 668–679. 10.1016/j.neuroimage.2018.12.046 30593903

[IMAG.a.1017-b26] Grootswagers, T., Robinson, A. K., Shatek, S. M., & Carlson, T. A. (2019b). Untangling featural and conceptual object representations. NeuroImage, 202, 116083. 10.1016/j.neuroimage.2019.116083 31400529

[IMAG.a.1017-b27] Grootswagers, T., Wardle, S. G., & Carlson, T. A. (2017). Decoding dynamic brain patterns from evoked responses: A tutorial on multivariate pattern analysis applied to time series neuroimaging data. Journal of Cognitive Neuroscience, 29, 677–697. 10.1162/jocn_a_01068 27779910

[IMAG.a.1017-b28] Grootswagers, T., Zhou, I., Robinson, A. K., Hebart, M. N., & Carlson, T. A. (2022). Human EEG recordings for 1,854 concepts presented in rapid serial visual presentation streams. Scientific Data, 9, 3. 10.1038/s41597-021-01102-7 35013331 PMC8748587

[IMAG.a.1017-b29] Güntekin, B., & Başar, E. (2014). A review of brain oscillations in perception of faces and emotional pictures. Neuropsychologia, 58, 33–51. 10.1016/j.neuropsychologia.2014.03.014 24709570

[IMAG.a.1017-b30] Harmony, T. (2013). The functional significance of delta oscillations in cognitive processing. Frontiers in Integrative Neuroscience, 7, 83. 10.3389/fnint.2013.00083 24367301 PMC3851789

[IMAG.a.1017-b31] Hebart, M. N., & Baker, C. I. (2018). Deconstructing multivariate decoding for the study of brain function. NeuroImage, 180, 4–18. 10.1016/j.neuroimage.2017.08.005 28782682 PMC5797513

[IMAG.a.1017-b32] Hickok, G. (2014). The myth of mirror neurons: The real neuroscience of communication and cognition. W W Norton & Co, New York, NY, US. 10.26439/persona2016.n019.981

[IMAG.a.1017-b33] Jasper, H. H. (1958). The ten twenty electrode system of the international federation. Electroencephalography and Clinical Neurophysiology, 10, 371–375. 10.1016/0013-4694(58)90051-8 10590970

[IMAG.a.1017-b34] Jeffreys, S. H. (1998). The theory of probability (Oxford Classic Texts in the Physical Sciences), Third Edition. Oxford University Press, Oxford, New York. 10.1093/gji.6.4.555

[IMAG.a.1017-b35] Kanwisher, N., Chun, M. M., McDermott, J., & Ledden, P. J. (1996). Functional imaging of human visual recognition. Cognitive Brain Research, 5, 55–67. 10.1016/S0926-6410(96)00041-9 9049071

[IMAG.a.1017-b36] Kass, R. E., & Raftery, A. E. (1995). Bayes factors. Journal of the American Statistical Association, 90, 773–795. 10.1080/01621459.1995.10476572

[IMAG.a.1017-b37] Keysers, C., & Gazzola, V. (2009). Expanding the mirror: Vicarious activity for actions, emotions, and sensations. Current Opinion in Neurobiology, 19, 666–671. 10.1016/j.conb.2009.10.006 19880311

[IMAG.a.1017-b38] Keysers, C., & Gazzola, V. (2006). Towards a unifying neural theory of social cognition. Progress in Brain Research, 156, 379–401. 10.1016/S0079-6123(06)56021-2 17015092

[IMAG.a.1017-b39] Knyazev, G. G. (2012). EEG delta oscillations as a correlate of basic homeostatic and motivational processes. Neuroscience & Biobehavioral Reviews, 36, 677–695. 10.1016/j.neubiorev.2011.10.002 22020231

[IMAG.a.1017-b40] Knyazev, G. G. (2007). Motivation, emotion, and their inhibitory control mirrored in brain oscillations. Neuroscience & Biobehavioral Reviews, 31, 377–395. 10.1016/j.neubiorev.2006.10.004 17145079

[IMAG.a.1017-b41] Kriegeskorte, N., & Kievit, R. A. (2013). Representational geometry: Integrating cognition, computation, and the brain. Trends in Cognitive Sciences, 17, 401–412. 10.1016/j.tics.2013.06.007 23876494 PMC3730178

[IMAG.a.1017-b42] Krizhevsky, A., Sutskever, I., & Hinton, G. E. (2012). ImageNet classification with deep convolutional neural networks. In Advances in neural information processing systems. Curran Associates, Inc. 10.1145/3065386

[IMAG.a.1017-b43] Lamm, C., Silani, G., & Singer, T. (2015). Distinct neural networks underlying empathy for pleasant and unpleasant touch. Cortex, 70, 79–89. 10.1016/j.cortex.2015.01.021 25725510

[IMAG.a.1017-b44] Lamme, V. A. F., & Roelfsema, P. R. (2000). The distinct modes of vision offered by feedforward and recurrent processing. Trends in Neurosciences, 23, 571–579. 10.1016/S0166-2236(00)01657-X 11074267

[IMAG.a.1017-b45] Lee Masson, H., & Isik, L. (2023). Rapid processing of observed touch through social perceptual brain regions: An EEG-fMRI fusion study. Journal of Neuroscience, 43, 7700–7711. 10.1523/JNEUROSCI.0995-23.2023 37871963 PMC10634570

[IMAG.a.1017-b46] Lee Masson, H., Pillet, I., Boets, B., & Op de Beeck, H. (2020). Task-dependent changes in functional connectivity during the observation of social and non-social touch interaction. Cortex, 125, 73–89. 10.1016/j.cortex.2019.12.011 31978744

[IMAG.a.1017-b47] Lee Masson, H., Van De Plas, S., Daniels, N., & Op de Beeck, H. (2018). The multidimensional representational space of observed socio-affective touch experiences. NeuroImage, 175, 297–314. 10.1016/j.neuroimage.2018.04.007 29627588 PMC5971215

[IMAG.a.1017-b48] Malach, R., Reppas, J. B., Benson, R. R., Kwong, K. K., Jiang, H., Kennedy, W. A., Ledden, P. J., Brady, T. J., Rosen, B. R., & Tootell, R. B. (1995). Object-related activity revealed by functional magnetic resonance imaging in human occipital cortex. Proceedings of the National Academy of Sciences of the United States of America, 92, 8135–8139. 10.1073/pnas.92.18.8135 7667258 PMC41110

[IMAG.a.1017-b49] Marsh, A. A. (2018). The neuroscience of empathy. Current Opinion in Behavioral Sciences, 19, 110–115. 10.1016/j.cobeha.2017.12.016

[IMAG.a.1017-b50] Martínez-Jauand, M., González-Roldán, A. M., Muñoz, M. A., Sitges, C., Cifre, I., & Montoya, P. (2012). Somatosensory activity modulation during observation of other’s pain and touch. Brain Research, 1467, 48–55. 10.1016/j.brainres.2012.05.055 22683688

[IMAG.a.1017-b51] McGlone, F., Wessberg, J., & Olausson, H. (2014). Discriminative and affective touch: Sensing and feeling. Neuron, 82, 737–755. 10.1016/j.neuron.2014.05.001 24853935

[IMAG.a.1017-b52] McMahon, E., Bonner, M. F., & Isik, L. (2023). Hierarchical organization of social action features along the lateral visual pathway. Current Biology, 33, 5035–5047.e8. 10.1016/j.cub.2023.10.015 37918399 PMC10841461

[IMAG.a.1017-b53] McMahon, E., Isik, L. (2023). Seeing social interactions. Trends in Cognitive Sciences, 27, 1165–1179. 10.1016/j.tics.2023.09.001 37805385 PMC10841760

[IMAG.a.1017-b54] Meltzoff, A. N., Ramírez, R. R., Saby, J. N., Larson, E., Taulu, S., & Marshall, P. J. (2018). Infant brain responses to felt and observed touch of hands and feet: An MEG study. Developmental Science, 21, e12651. 10.1111/desc.12651 29333688 PMC6045975

[IMAG.a.1017-b55] Moerel, D., Rich, A. N., & Woolgar, A. (2024). Selective attention and decision-making have separable neural bases in space and time. Journal of Neuroscience, 44(38), e0224242024. 10.1523/JNEUROSCI.0224-24.2024 39107058 PMC11411586

[IMAG.a.1017-b56] Morey, R. D., Romeijn, J.-W., & Rouder, J. N. (2016). The philosophy of Bayes factors and the quantification of statistical evidence. Journal of Mathematical Psychology, 72, 6–18. 10.1016/j.jmp.2015.11.001

[IMAG.a.1017-b57] Morey, R. D., Rouder, J. N., & Jamil, T. (2018). BayesFactor: Computation of Bayes factors for common designs. 10.32614/cran.package.bayesfactor

[IMAG.a.1017-b58] Nicolardi, V., Lisi, M. P., Mello, M., Fusaro, M., Tieri, G., & Aglioti, S. M. (2025). Taking an embodied avatar’s perspective modulates the temporal dynamics of vicarious pain and pleasure: A virtual reality and EEG study. Social Cognitive and Affective Neuroscience, 20(1), nsaf035. 10.1093/scan/nsaf035 40279172 PMC12068220

[IMAG.a.1017-b59] Oostenveld, R., & Praamstra, P. (2001). The five percent electrode system for high-resolution EEG and ERP measurements. Clinical Neurophysiology, 112, 713–719. 10.1016/S1388-2457(00)00527-7 11275545

[IMAG.a.1017-b60] Peelen, M. V., Atkinson, A. P., Andersson, F., & Vuilleumier, P. (2007). Emotional modulation of body-selective visual areas. Social Cognitive and Affective Neuroscience, 2, 274–283. 10.1093/scan/nsm023 18985133 PMC2566760

[IMAG.a.1017-b61] Peelen, M. V., & Downing, P. E. (2007). The neural basis of visual body perception. Nature Reviews Neuroscience, 8, 636–648. 10.1038/nrn2195 17643089

[IMAG.a.1017-b62] Peirce, J., Gray, J. R., Simpson, S., MacAskill, M., Höchenberger, R., Sogo, H., Kastman, E., & Lindeløv, J. K. (2019). PsychoPy2: Experiments in behavior made easy. Behavior Research Methods, 51, 195–203. 10.3758/s13428-018-01193-y 30734206 PMC6420413

[IMAG.a.1017-b63] Peled-Avron, L., Levy-Gigi, E., Richter-Levin, G., Korem, N., & Shamay-Tsoory, S. G. (2016). The role of empathy in the neural responses to observed human social touch. Cognitive, Affective & Behavioral Neuroscience, 16, 802–813. 10.3758/s13415-016-0432-5 27165338

[IMAG.a.1017-b64] Peled-Avron, L., & Woolley, J. D. (2022). Understanding others through observed touch: Neural correlates, developmental aspects, and psychopathology. Current Opinion in Behavioral Sciences, 43, 152–158. 10.1016/j.cobeha.2021.10.002

[IMAG.a.1017-b65] Pihko, E., Nangini, C., Jousmäki, V., & Hari, R. (2010). Observing touch activates human primary somatosensory cortex. European Journal of Neuroscience, 31, 1836–1843. 10.1111/j.1460-9568.2010.07192.x 20584188

[IMAG.a.1017-b66] Pitcher, D., & Ungerleider, L. G. (2021). Evidence for a third visual pathway specialized for social perception. Trends in Cognitive Sciences, 25, 100–110. 10.1016/j.tics.2020.11.006 33334693 PMC7811363

[IMAG.a.1017-b67] Rigato, S., Banissy, M. J., Romanska, A., Thomas, R., van Velzen, J., & Bremner, A. J. (2019a). Cortical signatures of vicarious tactile experience in four-month-old infants. Developmental Cognitive Neuroscience, 35, 75–80. 10.1016/j.dcn.2017.09.003 28942240 PMC6968956

[IMAG.a.1017-b68] Rigato, S., Bremner, A. J., Gillmeister, H., & Banissy, M. J. (2019b). Interpersonal representations of touch in somatosensory cortex are modulated by perspective. Biological Psychology, 146, 107719. 10.1016/j.biopsycho.2019.107719 31207259

[IMAG.a.1017-b69] Robinson, A. K., Grootswagers, T., & Carlson, T. A. (2019). The influence of image masking on object representations during rapid serial visual presentation. NeuroImage, 197, 224–231. 10.1016/j.neuroimage.2019.04.050 31009746

[IMAG.a.1017-b70] Rouder, J. N., Speckman, P. L., Sun, D., Morey, R. D., & Iverson, G. (2009). Bayesian t tests for accepting and rejecting the null hypothesis. Psychonomic Bulletin & Review, 16, 225–237. 10.3758/PBR.16.2.225 19293088

[IMAG.a.1017-b71] Schirmer, A., & McGlone, F. (2019). A touching Sight: EEG/ERP correlates for the vicarious processing of affectionate touch. Cortex, 111, 1–15. 10.1016/j.cortex.2018.10.005 30419352

[IMAG.a.1017-b72] Scholl, B. J., & Gao, T. (2013). Perceiving animacy and intentionality: Visual processing or higher-level judgment. In M. D. Rutherford & V. A. Kuhlmeier (Eds.), Social perception: Detection and interpretation of animacy, agency, and intention (pp. 197–229). Boston Review. 10.7551/mitpress/9780262019279.003.0009

[IMAG.a.1017-b73] Schupp, H. T., Öhman, A., Junghöfer, M., Weike, A. I., Stockburger, J., & Hamm, A. O. (2004). The facilitated processing of threatening faces: An ERP analysis. Emotion, 4, 189–200. 10.1037/1528-3542.4.2.189 15222855

[IMAG.a.1017-b74] Smit, S., Crossley, M. J., Zopf, R., & Rich, A. N. (2025a). Characteristics of vicarious touch reports in a general population. Scientific Reports, 15. 10.1038/s41598-025-03194-2 PMC12480766 41022807

[IMAG.a.1017-b75] Smit, S., Moerel, D., Zopf, R., & Rich, A. N. (2023). Vicarious touch: Overlapping neural patterns between seeing and feeling touch. NeuroImage, 278, 120269. 10.1016/j.neuroimage.2023.120269 37423272

[IMAG.a.1017-b76] Smit, S., Ramirez-Haro, A., Varlet, M., Moerel, D., Quek, G., & Grootswagers, T. (2025b). A comprehensive EEG dataset for investigating visual touch perception. bioRxiv. 10.1101/2025.07.10.664069 PMC12992785 41654515

[IMAG.a.1017-b77] Smit, S., & Rich, A. N. (2025). The validated touch-video database. Behavior Research, 57, 134. 10.3758/s13428-025-02655-w PMC11958466 40164957

[IMAG.a.1017-b78] Smit, S., Rich, A. N., & Zopf, R. (2019). Visual body form and orientation cues do not modulate visuo-tactile temporal integration. PLoS One, 14, e0224174. 10.1371/journal.pone.0224174 31841510 PMC6913941

[IMAG.a.1017-b79] Stecher, R., & Kaiser, D. (2024). Representations of imaginary scenes and their properties in cortical alpha activity. Scientific Reports, 14, 12796. 10.1038/s41598-024-63320-4 38834699 PMC11150249

[IMAG.a.1017-b80] Streltsova, A., & McCleery, J. P. (2014). Neural time-course of the observation of human and non-human object touch. Social Cognitive and Affective Neuroscience, 9, 333–341. 10.1093/scan/nss142 23202659 PMC3980802

[IMAG.a.1017-b81] Teichmann, L., Moerel, D., Baker, C., & Grootswagers, T. (2022). An empirically driven guide on using Bayes factors for M/EEG decoding. Aperture Neuro, 1, 1–10. 10.1101/2021.06.23.449663

[IMAG.a.1017-b82] Ward, J., & Banissy, M. J. (2015). Explaining mirror-touch synesthesia. Cognitive Neuroscience, 6, 118–133. 10.1080/17588928.2015.1042444 25893437

[IMAG.a.1017-b83] Wetzels, R., Matzke, D., Lee, M. D., Rouder, J. N., Iverson, G. J., & Wagenmakers, E.-J. (2011). Statistical evidence in experimental psychology: An empirical comparison using 855 t tests. Perspectives on Psychological Science, 6, 291–298. 10.1177/1745691611406923 26168519

[IMAG.a.1017-b84] Whitmarsh, S., Nieuwenhuis, I. L. C., Barendregt, H., & Jensen, O. (2011). Sensorimotor alpha activity is modulated in response to the observation of pain in others. Frontiers in Human Neuroscience, 5, 91. 10.3389/fnhum.2011.00091 22007165 PMC3188815

[IMAG.a.1017-b85] Whitmarsh, S., Oostenveld, R., Almeida, R., & Lundqvist, D. (2017). Metacognition of attention during tactile discrimination. NeuroImage, 147, 121–129. 10.1016/j.neuroimage.2016.11.070 27908789

[IMAG.a.1017-b86] Williams, L. M., Palmer, D., Liddell, B. J., Song, L., & Gordon, E. (2006). The ‘when’ and ‘where’ of perceiving signals of threat versus non-threat. NeuroImage, 31, 458–467. 10.1016/j.neuroimage.2005.12.009 16460966

[IMAG.a.1017-b87] Xie, S., Kaiser, D., & Cichy, R. M. (2020). Visual imagery and perception share neural representations in the alpha frequency band. Current Biology, 30, 2621–2627.e5. 10.1016/j.cub.2020.04.074 32531274 PMC7342016

[IMAG.a.1017-b88] Yang, C.-Y., Decety, J., Lee, S., Chen, C., & Cheng, Y. (2009). Gender differences in the mu rhythm during empathy for pain: An electroencephalographic study. Brain Research, 1251, 176–184. 10.1016/j.brainres.2008.11.062 19083993

