# Supplementary material for: Rapid visual engagement in neural processing of detailed touch interactions
Source: Imaging Neurosci (Camb). 2025 Nov 17;3:IMAG.a.1017. doi: 10.1162/IMAG.a.1017 (PMC12624366; doi:10.1162/IMAG.a.1017)
Supplement: Supplementary Material [file IMAG.a.1017_supp.pdf]

Supplementary Table 1: Correlations between ratings for the adapted videos used in this study and the original videos from the Validated Touch-Video Database.

Video Orientation	Touch Dimension	Correlation (r)	Bayes Factor
Normal	Neutral	0.88	59.69
Normal	Pleasant	0.95	92.41
Normal	Unpleasant	0.82	44.51
Normal	Painful	0.93	82.61
Normal	Threat	0.97	109.24
Normal	Arousal	0.88	61.02
Horizontal Flip	Neutral	0.86	54.11
Horizontal Flip	Pleasant	0.94	86.41
Horizontal Flip	Unpleasant	0.82	45.37
Horizontal Flip	Painful	0.91	70.01
Horizontal Flip	Threat	0.95	94.6
Horizontal Flip	Arousal	0.86	53.16
Vertical Flip	Neutral	0.85	51.08
Vertical Flip	Pleasant	0.93	82.16
Vertical Flip	Unpleasant	0.84	49.46
Vertical Flip	Painful	0.9	67.54
Vertical Flip	Threat	0.95	94.58
Vertical Flip	Arousal	0.87	55.73
Hor & Vert Flip	Neutral	0.83	45.8
Hor & Vert Flip	Pleasant	0.92	76.84
Hor & Vert Flip	Unpleasant	0.85	51.11
Hor & Vert Flip	Painful	0.9	65.38
Hor & Vert Flip	Threat	0.95	96.65
Hor & Vert Flip	Arousal	0.88	58.53
